# Trends in publication on evidence-based antioxidative herbal medicines in management of diabetic nephropathy

**DOI:** 10.1186/s40200-016-0221-2

**Published:** 2016-02-13

**Authors:** Ozra Tabatabaei-Malazy, Rasha Atlasi, Bagher Larijani, Mohammad Abdollahi

**Affiliations:** 1 Diabetes Research Center, Endocrinology and Metabolism Clinical Sciences Institute, Tehran University of Medical Sciences, Tehran, Iran; 2 Endocrinology and Metabolism Research Center, Endocrinology and Metabolism Clinical Sciences Institute, Tehran University of Medical Sciences, Tehran, Iran; 3 EBM Group, Endocrinology and Metabolism Research Center, Endocrinology and Metabolism Clinical Sciences Institute, Tehran University of Medical Sciences, Tehran, Iran; 4 Department of Toxicology and Pharmacology, Faculty of Pharmacy, and Pharmaceutical Sciences Research Center, Tehran University of Medical Sciences, Tehran, Iran

**Keywords:** Herbal medicine, Antioxidative, Diabetic nephropathy, Scientometric analysis

## Abstract

**Background:**

Recently, popularity and use of herbal medicine in treatment of diabetes have been increased. Since, oxidative stress is known as the main underlying pathophysiology of diabetes and its complications, the purpose of this bibliometric study is to assess the global scientific production analysis and developing its trend in field of antioxidative hypoglycemic herbal medicines and diabetic nephropathy focusing on the scientific publication numbers, citations, geographical distribution in the world and determining the main journal (source) in the field.

**Methods:**

Our search terms were “diabetes”, “renal”, “nephropathy”, “herb”, “Chinese medicine”, “traditional medicine”, and “antioxidant” from Scopus database until January 2015 and analysis of the distribution of words in the publication year, main journal (source) in the field, geographical distribution, documents’ type and language, subject area, and h-index of citations were crried out. The Scopus analysis tools and VOSviewer software version 1.6.3 have been used for analysis.

**Results:**

Within 1166 papers were published until year 2015, 78 studies were related to this topic in human. Increasing trend in number of related researches was shown. Fifty eight percent of the published papers were original articles, and the highest number was produced in 2013 with 21 documents. Top subject areas were medicine with global publication share of 71.8 %, and pharmacology was ranked the second (39.7 %). Iran was the first country with global publication. The total citation of the documents were 2518 times and h-index was 24. The highest cited paper was a review article with 336 citation number, and top source was “Journal of Medicinal Plants”. Both of top authors and affiliation were from Iran; “Tehran University of Medical Sciences”. Also, top author in the co-authorship mapping and clustering assessment was from Iran.

**Conclusions:**

Although, we found an ascending trend of scientific publications in field of antioxidative herbal medicine and diabetic nephropathy with a good position for Iran, the number of publications is insufficient and more researches in this topic is necessary.

## Background

The prevalence of Diabetes Mellitus (DM), as the most common cause of chronic kidney disease (CKD) and renal failure, is increasing worldwide. American Diabetes Association (ADA) reported 20-40 % of diabetic patients have suffered from diabetic nephropathy [1]. Therefore, CKD should be considered as one of the most common complication of diabetes, and early intervention to delay progression to diabetic nephropathy and CKD along with providing good glycemic control would be important. Although, many synthetic drugs are available in treatment of diabetic nephropathy, only a fraction of them can be used safely in these patients. In addition, due to undesirable side effects and high cost of synthetic drugs, their dosage should be adjusted in renal failure [2]. Thus, considering alternative treatments such as herbal medicines that have been determined their safety and efficacy in the management of diabetes and its complications would be logical. Recently, popularity and use of herbal medicine among different age groups in both developing and developed countries for treatment of diabetes have been increased [2–4]. However, there has been no systematic analysis of scientific productions’ trends in field of antioxidative hypoglycemic herbal medicines and diabetic nephropathy. The scientometric analysis has been determined as a reliable and practical method to measure, and evaluate the current scientific research directions in a specific field by focusing on numbers of published papers and their citation’ numbers [5]. Considering the increasing prevalence of diabetes and its complications, there exists a need for sufficient scientific evidences obtained from scientific interactions and journals to design a suitable preventive plan [2–4, 6]. In addition, it is more essential to pay attention to oxidative stress as the main underlying pathology of diabetes and its complications [7–10]. In the present study, we investigate a scientometric study of scientific productions in field of antioxidative herbal medicines and diabetic nephropathy focusing on the scientific publication numbers, citations, geographical distribution in the world, determining the main journal (source) in the field, and co-authorship mapping and clustering assessment.

## Methods

### Data source

The study is based on information from Scopus database for scientific publications and on growth data recorded in the literature. The Scopus database was known as one of the most comprehensive source of bibliographic data due to its high coverage in health and biomedicine disciplines and its reliable citations’ report for academic articles. In addition, it is important its accessibility to valid different analysis tools [11–13] that can feasible analysis of the collected data. The quality and reliability of the information gathered, in particular with regard to citation and author affiliations were considered.

### Search strategies

The Scopus database was searched to compile a bibliography of all papers related on antioxidative herbal medicines in management of diabetic nephropathy which compromising ((TITLE-ABS-KEY (“Phytotherapy” OR “Naturopathic” OR “Drug*” OR “Plant*” OR “Medic*” OR “Herb*” OR “Flower*”) AND TITLE-ABS-KEY ((“Chinese” OR “Traditional” OR “Primitive” OR “Indigenous”) OR (“Folk” AND “Remed*”) OR “Ethnomedicine” OR (“Remedy” AND “Home”) OR “Ethnopharmacology” OR “NATURAL” OR “extract*” OR “COMPONENT*”) AND TITLE-ABS-KEY(“DIABET*” AND “TYPE2” OR “type 2” OR “TYPEII” OR “TYPE II” OR “TYPE-II” OR “T2DM” OR “T2D” OR “NIDDM”) AND ALL(“Antioxidant*” OR “Anti-Oxidant” OR “Anti Oxidant” OR “Antioxidative” OR “Anti-Oxidative” OR “Anti Oxidative” OR “OXIDANT” OR “OXIDANTS” OR “OXIDATIVE”)) AND ALL(“kidney” OR “renal” OR “nephro*” OR “protein*” OR “albumin*” OR “creatin*”). The meaning of the TITLE-ABS-KEY code combined field is searching abstracts, keywords, and article titles.

The inclusion criteria were all relevant available scientific publications in field of antioxidative hypoglycemic herbal medicine and diabetic nephropathy which conducted in human and published before January 2015.

After assessment literature in title and abstract of all papers, duplicated articles were excluded. Other exclusion criteria were studies conducted in children, adolescents, pregnant women, type 1 diabetes mellitus, or animal studies. No language limitation was considered. Finally, 78 documents were analyzed.

### Data analysis

In overall, the impact factors (IF) of each journal that was used as a quantitative indicator to assess, compare, and rank of scientific publications [14], developed to faciliate comparison between citation rates of journals and evolved as a measurement of journal quality. The collected data were publication year, the main journal (source) in the field, author’s name and affiliation, country distribution, document’s type and language, subject area, documents’ citations, *h*-Index and the *h*-graph. The *h*-Index and *h*-graph are indicators to measure of research performance quality by showing the number of citations per document [15]. The Scopus web database was used to analysis extracted data. The SPSS version 15 was used to assess correlation between number of published papers and year of publication. VOSviewer (Visualizing Scientific Landscapes) software version 1.6.3 that is available in www.vosviewer.com has been used for co-authorship mapping and clustering assessment to provide information on scientific collaboration between co-authors in the field.

## Results

### Time-trend in publications

**Fig. 1:**
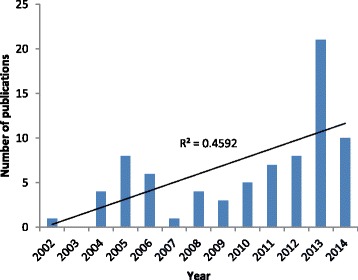
Time-trend distribution of document types in antioxidative herbal medicine and diabetic nephropathy

Figure 1 shows the distribution of document type identified by Scopus analyzed. It is shown three pikes in number of published articles in years 2005, 2008, and 2013 compared to previous years. We found the highest number of scientific productions in 2013 (26.9 % equivalent to 21 documents), while the lowest publication was in 2007, and 2002 (each one 1.28 %, equivalent to 1 document). The overall correlation reflecting the association between number of published documents and year of publication was 0.728 with *p* value = 0.005. The R-squared value of 0.459 suggests a steady and significant increase since 2002 to 2015.

### Type and language of documents

Our analysis reveals among 78 documents, the majority of them (45 documents, 57.7 %) were original articles, followed by review articles (33 documents, 42.3 %).

Totally, the studied documents were published in four languages; English, Persian, Chinese, and German. Most of documents were in English language (88.46 %).

### Subject area

Top subject area was medicine with global publication share of 71.8 percent. Pharmacology ranked the second (39.7 %) and biochemistry/genetics/molecular biology (20 documents, 25.6 %) was the third rank.

### Geographical distribution

In overall, in the global publication share of the top 5 countries, Iran topped the list with global publication share of 19 documents (25 %), the United States with 11 (14.47 %), China and India each one with 6 documents (7.89 %), and United Kingdom with 5 documents were 5 top countries with high number of publications.

### Citations’ numbers

**Fig. 2:**
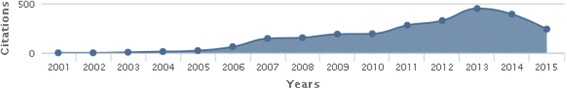
Chart of citation of published documents in our study

Figure 2 shows the changes in the total number of citations in each year. A total of 1166 publications were published until year 2015 have received to total number of citations 2518 times at the time of data analysis (until July 21^th^, 2015). The average number of citation per article was 33.13.

Among articles, 64 documents (82 %) were cited at least once and 14 documents (18 %) did not have any citation at all. The highest number of citations (336 times) was happen in 2006 while there was not any citations during years 2000 and 2002.

**Fig. 3:**
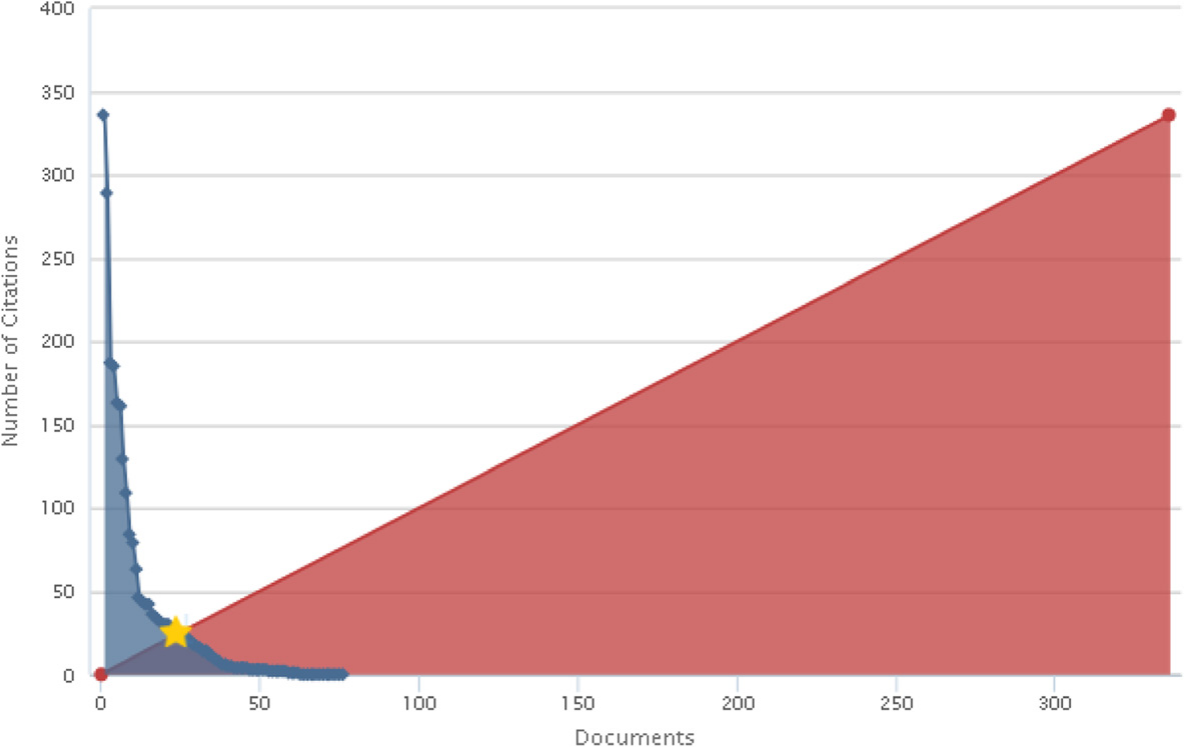
H-graph of published documents in the studied field

The *h*-index for 78 analyzed documents was 24. This means 24 documents were cited at least 24 times (Fig. 3).

In Table 1 that summarizes characteristics of the top ten highly cited articles, citation number ranged from 336 times to 79 times. Highly cited publication was a review paper entitled “Green tea and its polyphenolic catechins: medicinal uses in cancer and noncancer applications” which has been cited 336 times, followed by another review paper etitled “Curcumin: getting back to the roots”, 289 times. Findings shown that highly cited original article was entitled “Beneficial effects of soy phytoestrogen intake in postmenopausal women with type 2 diabetes” which has been cited 187 times.

**Table 1:** Citations and characteristics of top 10 highly cited published documents in antioxidative herbal medicine and diabetic nephropathy

Title	No. times cited	Year	Document type	Country	Journal (IF)	Subject area of the journal
Green tea and its polyphenolic catechins: medicinal uses in cancer and noncancer applications	336	2006	Review article	The United States	Life Sciences (2.702)	Biochemistry, general and molecular biology (Medicine)
Curcumin: getting back to the roots	289	2005	Review article	The United States	Annals of the New York Academy of Sciences (----)	Biochemistry, Genetics and Molecular Biology
Beneficial effects of soy phytoestrogen intake in postmenopausal women with type 2 diabetes	187	2002	Original article	United Kingdom	Diabetes Care (8.570)	Medicine
Antidiabetic agents from medicinal plants	185	2006	Review article	South Korea	Current Medicinal Chemistry (3.853)	-Molecular Medicine -Pharmacology
Resveratrol improves insulin sensitivity, reduces oxidative stress and activates the Akt pathway in type 2 diabetic patients	163	2011	Original article	The United States	British Journal of Nutrition (3.342)	Medicine
Mexican plants with hypoglycaemic effect used in the treatment of diabetes	161	2005	Review article	United Kingdom	Journal of Ethnopharmacology (2.998)	Pharmacology
Plant foods in the management of diabetes mellitus: Spices as beneficial antidiabetic food adjuncts	129	2005	Review article	India	International Journal of Food Sciences and Nutrition (1.206)	Food Science
Role of selected Indian plants in management of type 2 diabetes: a review	109	2004	Review article	India	Journal of Alternative and Complementary Medicine (1.585)	Medicine
Ginseng and diabetes	84	2005	Original article	The United States	American Journal of Chinese Medicine (2.625)	Medicine
The role of antioxidant micronutrients in the prevention of diabetic complications	79	2004	Review article	France	Treatments in Endocrinology (----)	Biochemistry, Genetics and Molecular Biology (Endocrinology)

Legend: *IF* Impact factor

As shown in Table 1, of 10 highly cited articles 7 articles were review, and 3 papers were original articles. The United States was ranked at first country with 4 highly cited papers which have the highest cited scientific publications. United Kingdom and India stand second rank.

In addition, we extracted sperately type of documents, type and number of highly cited study with name of the studied plant for each year (Table 2).

**Table 2:** Scientometry analysis of scientific productions on antioxidative hypoglycemic herbal medicines in diabetic nephropathy per year

Publication (yr)	Enrolled studies (*n*)	Type of studies			Highly cited study		
					Type of study	Citation (*n*)	Studied plant
		Original article (*n*)	Review article (*n*)	Case report (*n*)			
2014	10	7	3	----	Review article	12	Curcumin
2013	21	8	12	1	Review article	33	Polyphenols
2012	8	3	4	1	Review article	44	---- (natural products)
2011	7	5	2	-----	Original article	163	Resveratrol
2010	5	3	2	-----	Original article	30	*Satureja khuzestanica*
2009	3	3	-----	-----	Original article	63	Grape seed extract
2008	4	4	-----	-----	Original article	42	Pycnogenol
2007	1	-----	1	----	Review article	10	Flavonoid
2006	6	2	4	-----	Review article	336	Green tea
2005	8	2	6	-----	Review article	289	Curcumin
2004	4	1	3	-----	Review article	109	Indian plants
2002	1	1	------	----	Original article	187	Phytoestrogen

### Authors’ name & affiliation

We found 40 authors as the first-author that published papers in the field of hypoglycemic antioxidative herbal medicine and diabetic nephropathy. Table 3 shows the top 5 first-author that had the most of publications in the field. Among top 5 first-author, 4 authors were from Iran and one author was from Canada. Fallah Huseini, H.” with 11 articles followed by “Larijani, B.” with 6 papers had the highest number of publications in the studied field, and both of them were from Iran.

**Table 3:** Top 5 authors according to the highest number of published documents in our study

Rank	Author name	Documents (number/percent)	Affiliation	Country
1	Fallah Huseini, H./Huseini, H.F.	11/14.10	Iranian Academic Center for Education, Culture and Research, Department of Pharmacology and Applied Medicine	Iran
2	Larijani, B.	6/7.69	Tehran University of Medical Sciences, Endocrinology and Metabolism Research Center	Iran
3	Heshmat, R.	5/6.41	Tehran University of Medical Sciences, Chronic Diseases Research Center	Iran
4	Kianbakht, S.	5/6.41	Iranian Academic Center for Education, Culture and Research, Department of Pharmacology and Applied Medicine	Iran
5	Lee, T.	2/2.56	Health Centre of Milton, Milton, ON	Canada

### Co-authorship mapping & clustering

**Fig. 4:**
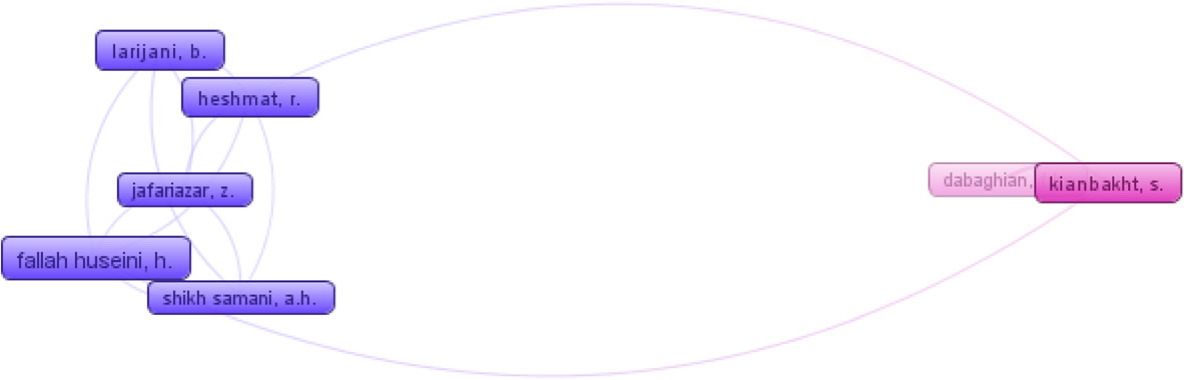
Map of co-authorship network of the authors published scientific papers in the studied field

**Fig. 5:**
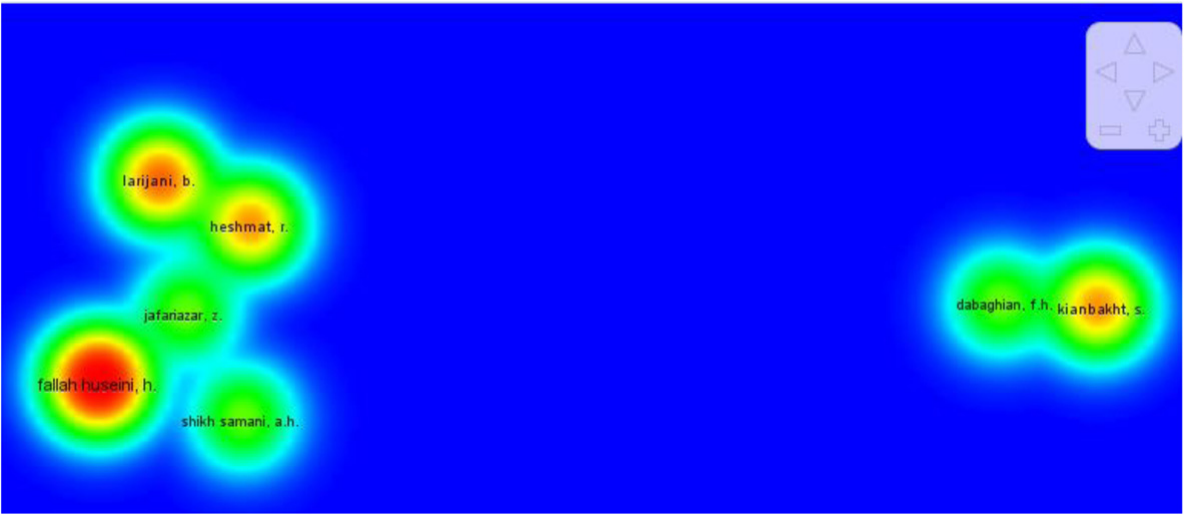
Density view of co-authorship network of the authors published scientific papers in the studied field

Co-authorship mapping and clustering assessment is one of the factors to provide information on scientific collaboration between co-authors in the field by using VOSviewer software. It was considered 2 documents as the minimum number of published scientific papers by one author to map co-authorship. Out of 342 authors, 11 authors meet this threshold. Within them, 2 auhors were without co-authorship which excluded from analysis. Co-authorship network and density views are shown in Figs. 3 and 4, respectively. Top 5 authors in our studied field based on co-authorship are “Fallah Huseini, H.” (16 co-authorship) followed by “Heshmat, R.” (13 co-authorship), “Larijani, B.” (11 co-authorship), “Kianbakht, S.” and “Jafariazar, Z.” each one with 7 co-auhorships (Fig. 4). According to Fig. 5, the highest density in the network belonged to “Fallah Huseini, H.”.

### Institutes’charastristics published documents

Table 4 shows the institution wise collaborations of publications with its affiliations. The Tehran University of Medical Sciences (TUMS)” has stood its first place with 13 publications (16.66 %). The second place has been occupied by Iranian Academic Center for Education, Culture and Research (ACECR)” with 10 published papers (12.82 %).

**Table 4:** Names and characteristics of top 5 institutes for the published documents in the studied filed

Rank	Institution name	Documents (number/percent)	Country
1	Tehran University of Medical Sciences	13/16.66	Iran
2	Iranian Academic Center for Education, Culture and Research	10/12.82	Iran
3	Iranian Ministry of Health and Medical Education	4/5.13	Iran
4	Baqiyatallah Medical Sciences University	3/3.85	Iran
5	Institute of Medicinal Plants Iran	3/3.85	Iran

In accordance with top 10 countries, all of 5 top institutions’ relations were from Iran. Details of the findings are shown in Table 4.

### Charastristics of main journal (source)

Table 5 shows the charastristics of main journals*.* The “Journal of Medicinal Plants” has scored the first rank with 7 publications. “Evidence Based Complementary and Alternative Medicine” with 5 documents stood second rank. Other ranks were followed by “Phytotherapy Research” with 3 documents, and “Fitoterapia” and “Complementary Therapies in Medicine” each one with 2 documents.

**Table 5:** Characteristics of top 5 sources for the published documents in field of antioxidative traditional medicine and diabetic nephropathy

Title of journal (IF)	Documents (number/percent)	Total citations to document	Citation per document	Citation to highest cited document	Plant studied in highest cited document
Journal of Medicinal Plants (----)	7/8.97	27	3.86	11	Anti-diabetic medicinal plants
Evidence Based Complementary and Alternative Medicine (1.88)	5/6.41	41	8.2	30	*Satureja khuzestanica*
Phytotherapy Research (2.66)	3/3.85	50	16.67	26	*Citrullus colocynthis (L.)*
Fitoterapia (2.216)	2/2.56	39	19.5	21	*Brown algae*
Complementary Therapies in Medicine (1.545)	2/2.56	6	3	3	- *Capparis spinosa L. (Caper) & Salvia officinalis L*

Legend: *IF* Impact factor

Impact factor of all of 5 top journals, except for “Journal of Medicinal Plants” were remarkable (nearly 2). Within studied herbal medicine in these journals, the highest cited document was on *Satureja khuzestanica*. Details of the results are shown in Table 5.

## Discussion

In the present study, 78 scientific products extraction was analyzed from Scopus database in field of antioxidative hypoglycemic plants and diabetic nephropathy.

Since, Scopus search engine is known as one of the largest abstract and citation database of peer-reviewed literature: scientific journals, books and conference proceedings [11–13], our study is given a reliable scientific visalization to researchers in published sholary literature performed in field of antioxidative hypoglycemic plants and diabetic nephropathy around the world.

Although, our findings showed an increasing trend in publishing the papers in studied field with a significant R-squared value of 0.459, we found some temporary declines in years’ 2006, 2007, 2009, and 2014. One reason of these reports may be related to negative effect of international sanction against Iran [16].

The majority of published products in the studied field were original articles (58 %). Due to WHO recommendation to encourage researchers for performing more scientific studies in field of herbal medicine and diabetes [17], we faced the increasing trend in the field of our study. In addition, when we assessed the subject area of the published documents, top subject area was medicine that followed by drug. As shown in Table 1, all of top 10 highly cited papers have published in international journals. There is a remarkable point here that these journals are not only scholarly valuable (IF > 2), but also the subject area of them are medicine and/or pharmacology. As we said previously, IF can reflect the importance of the paper with its number [14]. On the other word, journals with high IF are journals with high ranking. The *h*-index and *h*-gragh of our documents are revealed these top 10 highly cited articles are the highest quality papers, and so would be mostly published in the high-impact journals and/or seen by more readers due to availability of older articles for longer periods compared to more recent published papers. Accordingly, all of above our results are in line worldwide medical professions’ interest to discover new drugs that have more safety, less side effects and cost by focusing on natural products such as hypoglycemic antioxidative herbal medicines [2, 18].

After considering the citation report according to the article, the top document was entitled “Green tea and its polyphenolic catechins: medicinal uses in cancer and noncancer applications” that published in journal of “Life Scinces” with IF of 2.702 and 336 citation number. It noted from Scopus analysis tools’ report, this top document was a review article. The top 3 plant highly cited papers in plant in the present study were *Green tea*, *Curcumin*, and phytoestrogens. These studies were top 3 highly cited documents not only when analyzed total enrolled studies, but also when analyzed studies’ results sperately for each year. This fact could confirm our findings that we faced with growing interest of the medical professions to WHO recommendation.

According to country distribution of scientic publications in our study, the majority of the papers were from Iran and the the United States ranked the second. Iran’s 20 year national vision document was predicted the highest rank in science and technology for Iran compared to other developed country by 2025 [19]. Based on this, a high rate of published scholarly paper was expected from Iran. The first four authors of top 5 first-authors were Iranian which this fact was in line previous studies [20]. This fact was also approved by co-authorship mapping and clustering. In addition, when we assessed the institutional affiliation of the authors participated in publishing documents in the studied field, all of top 5 institutes were from Iran. “Tehran University of Medical Sciences” had the highest affiliation among institutes. On the other word, despite international sanction against Iran, the results of our study verified that Iran has a remarkable position in scientific productions in field of hypoglycemic antioxidative herbal medicine and diabetic nephropathy compared to other countries. The reason of this fact may be related to significantly increase in number of related multidisplinary faculties, research centers/institutes, related academic specialists, students, and research projects that all of them have been shown positive effects on number of published articles, and their citations [21].

Our study had some strengths as well as some limitations. Firstly, for the first time, this study focused on scientometric analysis of antioxidative hypoglycemic herbal medicine in diabetic nephropathy. Moreover, we used Scopus web database that has a high coverage in multidispline branches of science which can result in reliable analysis. The most limitation of this study was exclusion of scholarly literatures indexed outside of Scopus database that could be resulted in lose some highly cited scientific documents published in non-Scopus journals.

## Conclusions

The study concluded that there is an increasing trend in producing of scientific papers in field of antioxidative hypoglycemic herbal medicines and diabetic nephropathy, along remarkable position for Iranian scientists in this field. However, despit a significant promising in scientific productivity in the studied field, number of publications are insufficient and we need more researches and scholary publications in this topic.

### Availability of data and materials

As this study was a desk research which did not included any human subjects (neither human data nor human material), any ethical committee approval was needed. Moreover, for the same reason there was not necessary informed consent of the participants in the study. So, all of above three sections do not compatible.

### Endnotes

There is not any endnotes in the manuscript.

## Abbreviations

DM: Diabetes mellitus
CKD: Chronic kidney disease
ADA: American Diabetes Association
MESH: Medical subject headings
IF: Impact factor
VOSviewer: Visualizing Scientific Landscapes
TUMS: Tehran University of Medical Sciences
ACECR: Academic Center for Education, Culture and Research
